# Photodissociation of ClNO in the 2 ^1^A′ State: Computational and Experimental NO Product State Distributions

**DOI:** 10.1002/cphc.201200999

**Published:** 2013-03-07

**Authors:** Kiera M Jones, Jadwiga A Milkiewicz, Benjamin J Whitaker, Alan G Sage, Graham A Worth

**Affiliations:** [a] School of Chemistry University of LeedsLeeds LS2 9JT (UK); [b] School of Chemistry University of BristolBristol (UK); [c] School of Chemistry University of BirminghamBirmingham (UK)

**Keywords:** ab initio calculations, laser chemistry, nitrosyl chloride, photochemistry, velocity map imaging

## Abstract

Ultrafast photodissociation of the 2 ^1^A′ state of ClNO, which has an absorption spectrum peaking at 335 nm, is studied by computational and experimental methods. New potential-energy surfaces are calculated for the 1 and 2 ^1^A′ states at the multireference configuration interaction (MRCI) level. Wavepacket dynamics simulations performed both exactly and by using the multiconfiguration time-dependent Hartree method yield essentially identical results. Transition dipole moments at a range of geometries are included in these calculations to correctly model the excitation. Vibrational and rotational state distributions of the NO product are obtained both computationally by analysing the quantum flux on the 2 ^1^A′ surface and experimentally by use of 3D resonant multiphoton ionisation (REMPI), a variant of the velocity map imaging technique. The nascent NO is found to be only marginally vibrationally excited, with 91 % formed in *v*=0. The calculated NO rotational distribution peaks in the *j*=45–55 region, which compares favourably to experiment.

## 1. Introduction

The photodissociation of nitrosyl chloride (ClNO) has been studied as a prototypical example of bond rupture since the 1930s.[1] In the UV/Vis region (2–7 eV) ClNO exhibits strong absorption and readily dissociates to give NO and Cl products in their ground electronic states. The NO can be formed in a variety of vibrational and rotational states depending on the photon energy and hence the excited electronic state accessed.[2] With only 32 electrons it is amenable to computational study by ab initio methods. As an easily prepared, albeit highly corrosive, gas it is also convenient for detailed experimental investigation by techniques such as velocity map imaging. ClNO is also of interest to the atmospheric chemistry community, because it is a source of the Cl radical particularly in urbanised regions of the coast.[3] A further motivation for revisiting the photochemistry of this molecule from a computational perspective is the possibility of using it as a target molecule for coherent control experiments through optical pulse shaping.^[^^4^^,^ ^5^^]^

ClNO belongs to the *C_s_* point group and hence its electronic states are of A′ or A′′ symmetry. Ignoring spin–orbit coupling, there are 12 states which correlate to the ground electronic states of NO and Cl (^2^Π and ^2^P, respectively) on dissociation: 3×^1^A′, 3×^1^A′′, 3×^3^A′ and 3×^1^A′′. All of these barring 1 ^1^A′ are dissociative, but there is some variation in their lifetimes, with 1 ^1^A′′ and 1 ^3^A′′ in particular existing long enough to exhibit structured absorption bands.^[^^6^^–^^8^^]^ For the 2 ^1^A′ state of interest here the dissociation process is over more quickly, but the topology of the potential-energy surface (PES) still has a marked influence upon the NO product state distribution.

The 2 ^1^A′ state absorbs light between 280 and 400 nm (3 and 4.5 eV) with a peak at 335 nm (3.72 eV).[9] It is commonly referred to in the literature as the B band following the nomenclature introduced by Goodeve and Katz.[10] The exact identity of the state giving rise to this band was uncertain for some time, despite early calculations[11] and experimental work showing that it was of A′ symmetry.^[^^12^^,^ ^13^^]^ The problem was solved when Reisler and co-workers conducted photofragment yield spectroscopy experiments^[^^9^^,^ ^13^^]^ which confirmed the symmetry and showed it gave rise to NO fragments primarily in the Π(A′′) Λ-doublet state. In a Hartree–Fock molecular orbital model, this implied that excitation was to an orbital perpendicular to the plane of the molecule. Combining this information with ab initio calculations showed clearly that the 2 ^1^A’ state was responsible, with primary excitation occurring from the Cl p_*z*_ orbital to a NO π_*z*_ antibonding orbital. This assignment was further confirmed by later, more advanced calculations.^[^^14^^,^ ^15^^]^

The experiments of Reisler et al. also revealed the NO (*v*=0) rotational distributions following photodissociation at 355 nm, which were found to be bell-shaped, peaking at *j*≈46[13] or *j*≈43,[9] where *j* is the rotational quantum number of the NO nuclear frame. More recent, detailed state-selected experiments carried out by Torres et al. found a similar narrow distribution peaking at *j*=46 for NO in its ground vibrational state.[16] This high level of rotational excitation implies anisotropy on the excited-state surface, an observation borne out by previous calculations of the 2 ^1^A′ surface.[15]

Herein, vibrational and rotational distributions in the NO fragment following dissociation on the 2 ^1^A′ surface are calculated and compared to experimental distributions measured by using the 3D resonant multiphoton ionisation (REMPI) technique. The article also sets the foundation for forthcoming work on ClNO excited state surfaces including spin–orbit coupling, and on coherent control through optical pulse shaping.

## Methodology

Potential-Energy Surfaces: All electronic structure calculations were carried out by using the MOLPRO 2010 package.[17] Firstly, PESs for the 1 and 2 ^1^A′ states were constructed. 2464 ab initio points were calculated across the following binding coordinates:

1. *r*_NO_: every 0.2 bohr from 1.75 to 2.95 bohr.
2. *r*_ClN_: every 0.25 bohr from 2.75 to 6.0 bohr and then every 0.5 bohr to 10.0 bohr.
3. Bond angle *θ*: every 10° from 20 to 170°.

The initial orbitals were obtained by using the CASSCF method^[^^18^^,^ ^19^^]^ state-averaged over the first three states of ^1^A′ character. A full valence active space was used with the N and O 1s and Cl 1s, 2s and 2p orbitals kept doubly occupied but not frozen. For *r*_ClN_ values greater than 6.0 bohr the occupation of the molecular orbital corresponding to the Cl 3s atomic orbital had to be restricted to two to prevent erroneous orbital reordering.

The CASSCF orbitals were then used to calculate the 1 and 2 ^1^A′ states by the multireference configuration interaction (MRCI) method (single and double excitations).[20] The contribution of quadruple excitations to the overall energy was estimated by the Davidson correction with relaxed reference functions. All generated configuration state functions were included. For these calculations the Dunning augmented correlation consistent polarised valence quadruple zeta (aug-cc-pVQZ) basis set was used.

Although alternative fitting functions were investigated, the quality of the ab initio points was sufficiently good that the surfaces could be smoothly fitted by using 3D cubic splines. The resultant surfaces were used for dynamics calculations.

Wavepacket Dynamics Methods: The majority of the quantum dynamics calculations were performed by using the multiconfiguration time-dependent Hartree method (MCTDH)^[^^21^^,^ ^22^^]^ as implemented in the Heidelberg software package.[23] Although ClNO is a small molecule and can be treated by exact calculations with relative ease, the MCTDH method was chosen to allow many wavepacket propagations to be run rapidly with very little loss of accuracy, and also to allow the full functionality of the suite of analysis programs to be utilised. A full description of the MCTDH method is given in ref. [24].

Briefly, the MCTDH wavefunction is written as a sum of Hartree products, in which each degree of freedom is represented by single particle functions (SPFs). These SPFs are time-dependent basis functions, wherein lies the strength of the method: if the functions are allowed to adapt during a wavepacket propagation fewer are needed for an accurate description of the dynamics. The underlying equation of the MCTDH method is [Eq. (1)]:

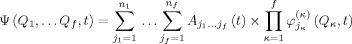
(1)
where *Q*_1_,..,*Q_f_* are the nuclear coordinates, 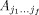 the expansion coefficients and 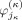 the SPFs for each degree of freedom *κ*. When more than one PES is included in the calculation multiple, distinct sets of these SPFs can be used for each surface.

Each SPF comprises a linear combination of time-independent functions known as the primitive basis. The form of the primitive basis functions is dependent upon the nature of the corresponding degree of freedom. To improve computational efficiency the discrete variable representation (DVR) was used for these, allowing the wavefunction to be localised onto a grid.[25] In this work, a Legendre DVR was chosen for the angular coordinate, with a sine DVR (using the particle-in-a-box functions as its basis) for the dissociative coordinate and a harmonic oscillator (HO) DVR for the bound coordinate.

**Figure 1:**
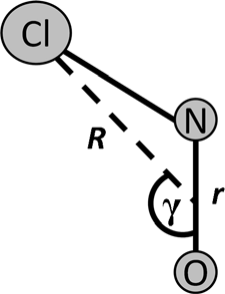
The Jacobi coordinates used in this work: *r* is the NO bond length, *R* the radial distance from the Cl atom to the centre of mass of the NO moiety and *γ* the angle between *r* and *R*, also known as the Jacobi scattering angle.

Although the PESs were calculated with binding coordinates, in the dynamics calculations they were converted to the Jacobi scattering coordinates shown in Figure 1. The main advantage of this coordinate system is that it leads to a considerable simplification of the kinetic energy operator. For systems in which the total angular momentum is taken to be zero (an approximation used throughout in this work), the operator is [Eq. (2)]:

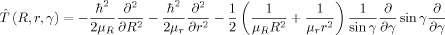
(2)

where *μ_R_* and *μ_r_* are the reduced masses for the *R* and *r* coordinates respectively.

After the PESs have been fitted by splines they are transformed into MCTDH product form by using the POTFIT program from the Heidelberg MCTDH suite.^[^^26^^,^ ^27^^]^ This allows the entire Hamiltonian to be written as a sum of products of SPFs and thus facilitates the calculation of the required integrals.

In the asymptotic region a complex absorbing potential (CAP)^[^^28^^,^ ^29^^]^ is placed on the *R* coordinate in order to absorb the wavepacket. This function has the form [Eq. (3)]:

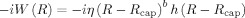
(3)
where *R*_cap_ is the start point, *η* is the strength (set here to 0.3), *b* is set to 3 and *h*(*R*−*R*_cap_) is a Heaviside step function. Without a CAP there is a danger that the wavepacket would hit the edge of the grid and be reflected backwards or reappear at the opposite end of the PES. In addition, it is very useful, as the quantum flux which passes into it can be recorded and subsequently analysed.

A standard method for obtaining product state distributions following wavepacket propagations is to take the time-dependent wavefunction in the asymptotic region and then project it onto the eigenstates of the individual product species. This can also be done by using the combination of the flux operator and the CAP, as outlined in ref. [30]. The flux operator 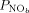 measures the quantum flux passing into the asymptotic region of the surface along the Jacobi *R* coordinate [Eq. (4)]:

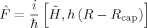
(4)
where *R*_cap_ is defined as before, and H̃ = Ĥ in order to include the CAP in the operator.

To calculate the scattering matrix from a ClNO photodissociation channel *a* into a NO product state *b* using this flux operator approach, the working equations are Equations (5) and (6):

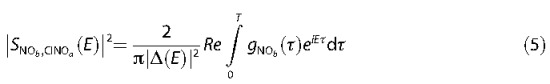
(5)

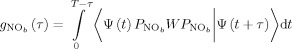
(6)
where *T* is the final propagation time, Δ(*E*) the energy spread of the initial wavepacket and 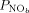 the projector onto the *b* state of NO. The form of the projector depends upon the state in question. It is also possible to apply multiple projectors, for example, to find out the amount of NO produced in a particular rotational state and a particular vibrational state.

In the exact calculations the wavepacket was propagated by using the short iterative Lanczos (SIL) method. In the MCTDH calculations the SIL method was used for propagating the expansion coefficients, and the Bulirsch–Stoer extrapolation integrator was used for the SPFs.[24]

Velocity Map Imaging: Velocity map imaging (VMI) is a well-established experimental methodology for recording quantum state selected product distributions following photodissociation.[31] Usually a nanosecond UV laser pulse is used to dissociate the molecule of interest, and a second pulse subsequently probes the products by REMPI. The resultant ions are then detected on a position-sensitive detector which, by virtue of the electrostatic lens used to extract them, provides a velocity (speed and direction) map of the state-selected photoproducts. In favourable circumstances a single laser pulse can act both to dissociate the target molecules and to ionize the desired photoproduct, which is the approach used here.

The apparatus used has been described elsewhere.^[^^32^^,^^33^^]^ Briefly, gaseous ClNO was prepared by mixing samples of Cl_2_ and NO in a stainless steel cylinder and backfilling with He carrier gas. Typical samples consisted of about 0.133 bar of Cl_2_ and about 0.266 bar of NO in 8.0 bar of He (≈5 %). The sample at a backing pressure of a few bar is expanded into a vacuum chamber through a pulsed valve (Parker, Iota one, series 9) and the resulting molecular beam is skimmed by a 1 mm-diameter skimmer (Molecular Beam Inc.) a few centimetres downstream of the valve through which it enters the ionization chamber. The velocity map electrostatic lens is configured on axis. Photodissociation was initiated by using a nanosecond laser pulse (Continuum Surelite II pumped Sirah Cobra-Stretch dye laser operating with Pyridine 2 or Styryl 8 and frequency-doubled) to a wavelength in the region of 350–380 nm.

The product NO was ionised by (2+1) REMPI via the D^2^Σ^+^←X^2^Π or C^2^Π←X^2^Π states using the same laser pulse. The frequency-doubled output of the dye laser (ca. 5 mJ) was focused onto the molecular beam in the centre of the ion optics by a 250 mm fused-silica lens. Using the two photon D^2^Σ^+^←X^2^Π and C^2^Π←X^2^Π transitions to detect NO has three advantages. Firstly, these transitions lie at convenient energies which allow photodissociation of the 2 ^1^A′ state to be studied in a single laser experiment. Secondly, unlike the more usual (1+1) REMPI probe of NO via the A^2^Σ^+^←X^2^Π band, the transitions are not easily saturated, and this makes it easier to quantify alignment effects. Thirdly, the Λ doubling of the NO(X^2^Π) rotational states is easily spectrally resolvable in the C^2^Π←X^2^Π band.

To gain a rapid overview of the rotational state product distribution and to easily discriminate between NO that might be present in the molecular beam as a contaminant from incomplete reaction or as a photoproduct of NO_2_ dissociation (another potential contaminant species), the velocity-resolved or 3D REMPI technique recently introduced by Dick and co-workers has been used.[34] In our version of the method, the voltages of the VMI lens are adjusted for direct-current slice-imaging,[35] and the microchannel plate detector is gated with a short (<10 ns) high-voltage pulse to record only a thin slice through the centre of the Newton sphere of state-selected ions, as described in ref. [33].

Our image processing software (in-house code written in LabView) works by recording a series of controided images as a function of wavelength, which are subsequently post-processed. Recording only the centroid coordinates rather than the entire image at each wavelength considerably reduces the data-storage requirements, and typically a 2 nm-wavelength scan with images recorded every 0.005 nm requires about 300 kb of disc space, as opposed to the 1 Gb or more needed for a sequence of uncompressed images. Radial integration of each of these images yields a 1D data array of intensity against pixel number, which is directly proportional to the velocity of the detected photofragment. Combination of many of these processed images as a function of wavelength yields a 3D “map” of wavelength, velocity and intensity. A typical experimental scan involved accumulating data for 150 laser shots per wavelength step.

## 2. Results

### 2.1. Surfaces

#### 2.1.1. 1 ^1^A′ Surface

The 1 ^1^A′, or S_0_, surface is the ground state of the ClNO molecule. The minimum-energy geometry calculated at the MRCI/aug-cc-pVQZ level of theory has *r*_ClN_=3.74 bohr and *r*_NO_=2.15 bohr with a bond angle *θ* of 113.4°. This is in very good agreement with the experimental optimum geometry of *r*_ClN_=3.72 bohr, *r*_NO_=2.14 bohr and *θ*=113.4°.^[^^36^^,^ ^37^^]^ The dissociation energy, obtained by taking the difference between the energies at the 1 ^1^A′ minimum and in the asymptotic region (with the same *r*_NO_ and *θ* values but with *r*_ClN_=20.0 bohr), is 1.619 eV. This again agrees very well with the experiment-based value of 1.615 eV.^[^^38^^,^ ^7^^]^

**Figure 2:**
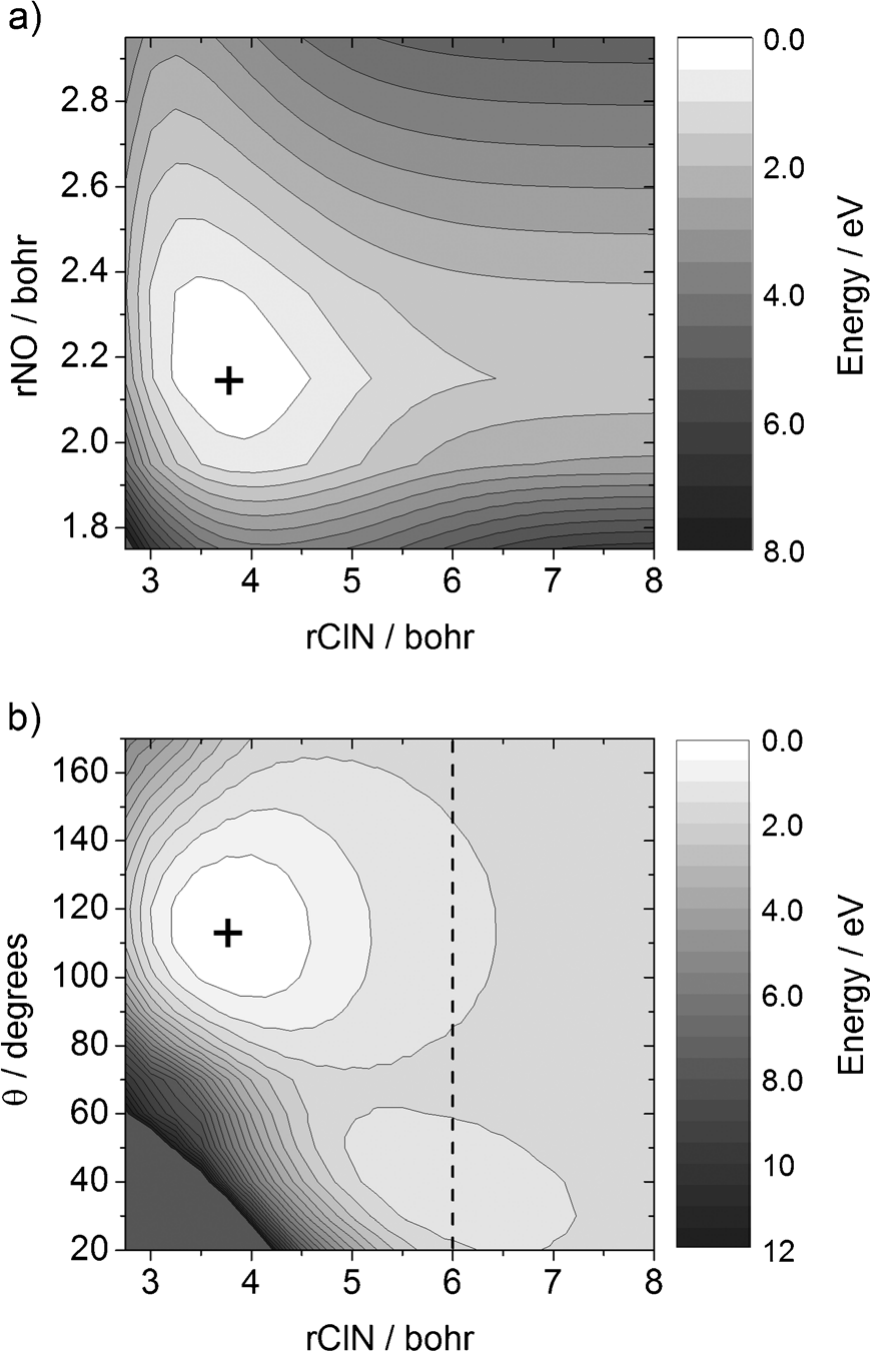
a) A cut through the 1 ^1^A′ surface at a constant angle *θ* of 110°, scanning the *r*_ClN_ and *r*_NO_ bond coordinates. b) A cut through the 1 ^1^A′ surface at a constant *r*_NO_ bond length of 2.15 bohr, scanning the *θ* and *r*_ClN_ coordinates. The dashed line marks the position of the 1D cut shown in Figure 3. Contour lines are given every 0.5 eV relative to the energy minimum. The crosses mark the minimum-energy geometry in each panel.

**Figure 3:**
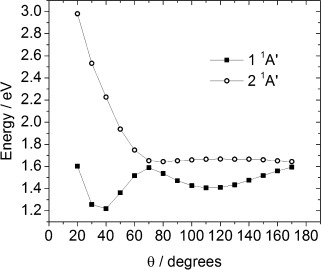
A cut through the 1 (squares) and 2 (circles) ^1^A′ surfaces close to the asymptotic region at a constant *r*_NO_ value of 2.15 bohr and a constant *r*_ClN_ value of 6 bohr. Note the interaction between the two states around *θ*=70°.

A cut through the surface at *θ=*110° is shown in Figure 2 a. Note that the well is not uniform, but instead extends slightly into the *r*_NO_ coordinate at lower *r*_ClN_ values. Figure 2 b shows a cut for *r*_NO_=2.15 bohr. The key features here are the two wells, one of which is at the Franck–Condon (FC) geometry and the other further out, centred at about *r*_ClN_=5.7 bohr, *r*_NO_=2.1 bohr and *θ*=40°, which corresponds to the ClON isomer.[39] This second well arises due to an interaction with the 2 ^1^A′ state occurring around *θ*=70° throughout the surface. The states are extremely close in energy at this point from about *r*_ClN_=5.75 bohr outwards for an *r*_NO_ value of 2.15 bohr. A one-dimensional cut at *r*_NO_=2.15 bohr and *r*_ClN_=6 bohr is given in Figure 3 to illustrate this. Work is underway to calculate the non-adiabatic coupling matrix elements between the two states in this region. Notably, it is rather unusual to find conical intersection seams in asymptotic regions, and this may be of significance in discussions of roaming dissociation mechanisms.

#### 2.1.2. 2 ^1^A′ Surface

The calculated energy difference between the 1 and 2 ^1^A′ states at the FC point is 3.704 eV, which is in good agreement with the experimental peak of the B band at 3.72 eV.[9] The energy difference between the dissociation asymptote and the FC point on the 2 ^1^A′ surface is −2.085 eV.

**Figure 4:**
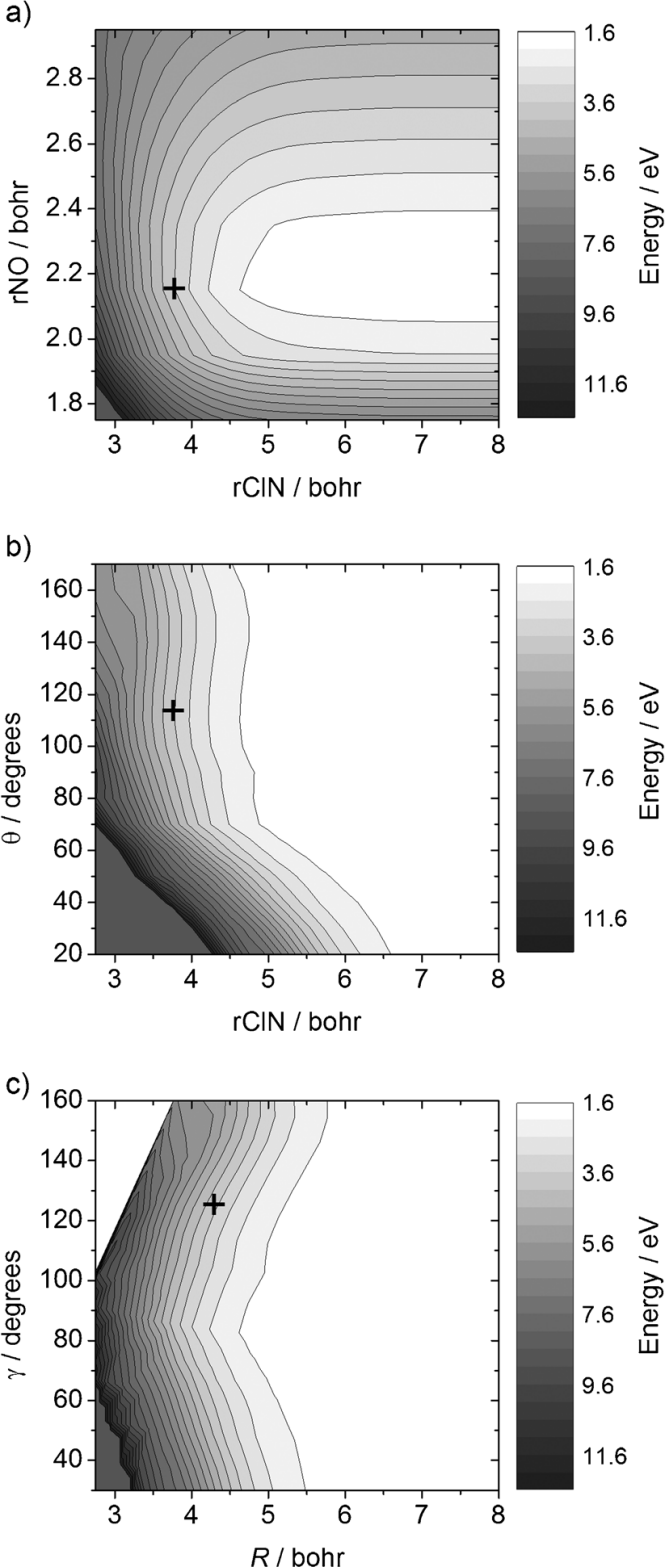
a) A cut through the 2 ^1^A′ surface at a constant angle *θ* of 110°, scanning the *R*_ClN_ and *r*_NO_ bond coordinates. b) A cut through the 2 ^1^A′ surface at a constant *r*_NO_ of 2.15 bohr, scanning the *θ* and *r*_ClN_ coordinates. c) A cut through the 2 ^1^A′ surface at a constant *r*_NO_ of 2.15 bohr scanning the Jacobi coordinates *γ* and *R* (as defined in Figure 1). Contour lines are given every 0.5 eV relative to the asymptotic energy of 1.6 eV. The crosses mark the approximate FC point in each panel.

Cuts through the 2 ^1^A′ surface are shown in Figure 4. A cut at *θ*=110° (Figure 4 a) shows the considerable steepness of the slope from the FC point to asymptotic regions. The cut at *r*_NO_=2.15 (Figure 4 b) shows clearly the kink at an angle of about *θ*=70° due to the intersection with the 1 ^1^A′ state. In Figure 4 c, the equivalent cut plotted in Jacobi coordinates shows how the slope of the surface at the FC point points steeply towards smaller *γ*.

#### 2.1.3. Transition Dipole Moments

The transition dipole moments (TDMs) between the two states were also calculated at each geometry at the MRCI level of theory. The TDMs calculated with MOLPRO comprise two perpendicular in-plane components aligned along the eigenvectors of the inertia tensor matrix. This gives a body-fixed view. Here, however, instead of considering the two in-plane components separately, they were treated together by calculating their resultant.

Most of these resultants lie along the Jacobi *R* coordinate. This is expected, as the excitation from the 1 ^1^A′ to the 2 ^1^A′ states involves promotion of an electron from a Cl 3p orbital to an NO π* orbital. For example, at the FC point, the component of the TDM aligned in the same direction as the Jacobi *R* coordinate is 0.076 a.u., and the component in the perpendicular coordinate is −0.0071 a.u. These FC values compare reasonably well with the results of previous calculations.[15]

**Figure 5:**
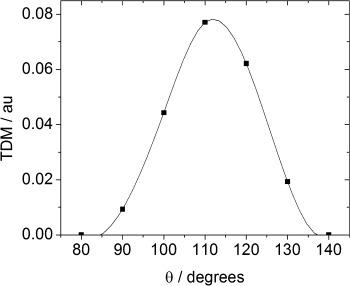
The resultants of the TDM components (given in atomic units) in the molecular plane at various angles for a fixed *r*_NO_ value of 2.15 bohr and a fixed *r*_ClN_ value of 3.75 bohr. The connecting line is a spline fit to guide the eye.

Calculated TDMs are rarely reliable outside of the FC region. In this work, they were found to vary smoothly across a limited region of the PES, from approximately *r*_ClN_=3.5 to 5.0 bohr and from *θ*=90 to 130°. Outside this region the TDMs were set to zero. For example, the cut in Figure 5 shows how the magnitudes of the TDMs vary with bond angle *θ*. The TDMs at each geometry were combined to create a “surface”, which was then converted to Jacobi coordinates and used in the dynamics calculations.

### 2.2. Wavepacket Dynamics

Quantum dynamics calculations were carried out to obtain information about the dissociation process in the 2 ^1^A′ state. The Hamiltonian used was given by Equation (7):

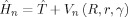
(7)
where T̂ is the kinetic energy operator stated in Equation (2) and *V_n_* is the PES for the desired state *n*. On the DVRs, 288 grid points were used in the *R* coordinate, 48 in the *r* coordinate and 200 in the *γ* coordinate. Calculations were run both exactly and by using the MCTDH method, and the two approaches gave near-identical results. For the MCTDH calculations, which are discussed here, ten SPFs were used for each coordinate.

Firstly, the wavepacket was relaxed on the 1 ^1^A′ surface to obtain the lowest-energy eigenstate for the system. This was achieved by propagating the wavepacket in imaginary time *iτ*[24] for a few tens of femtoseconds until there was no further change. Secondly, the wavepacket on the 2 ^1^A′ surface was formed by operating on the relaxed 1 ^1^A′ wavefunction using the TDM surface. Thirdly, the wavepacket on the excited state was allowed to propagate. A CAP was placed on the *R* coordinate at *R*=7.5 bohr to prevent reflection of the wavepacket from the edge of the grid.

**Figure 6:**
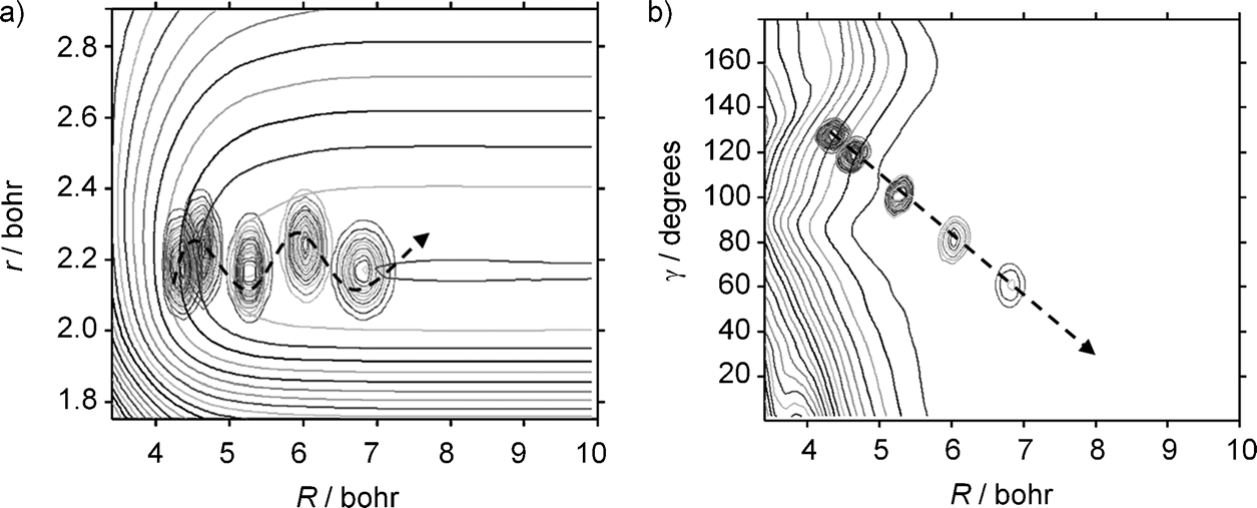
Snapshots of wavepacket propagation on the 2 ^1^A′ state of ClNO for a constant *γ* value of 127° (a) and for a constant *r*_NO_ value of 2.15 bohr (b). Jacobi coordinates are used. The wavepackets are shown at 0, 10, 20, 30 and 40 fs after excitation. The dotted arrows reflect the path of the wavepackets. Contour lines on the surfaces are 0.5 eV apart.

Snapshots of the wavepacket every 10 fs as it propagates on the 2 ^1^A′ surface are shown in Figure 6. As can be seen in the cut at *γ*=113° in Figure 6 a, the wavepacket receives only a small impetus in the NO coordinate, as its starting position is very close to the energy minimum. The NO product would therefore be expected to have little to no vibrational excitation. The cut at *r*_NO_=2.15 bohr shown in Figure 6 b is more interesting: the wavepacket is initially high on a steep wall angled towards lower *γ*, and thus proceeds rapidly downhill in this direction. The NO product would therefore be expected to have significant rotational excitation.

One of the most basic, and yet most revealing, pieces of data that can be obtained from such calculations is the autocorrelation function [Eq. (8)]:

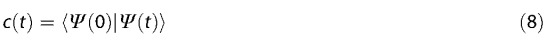
(8)
from which the total absorption spectrum is obtained by Fourier transformation [Eq. (9)]:^[^^40^^,^ ^41^^]^

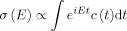
(9)

**Figure 7:**
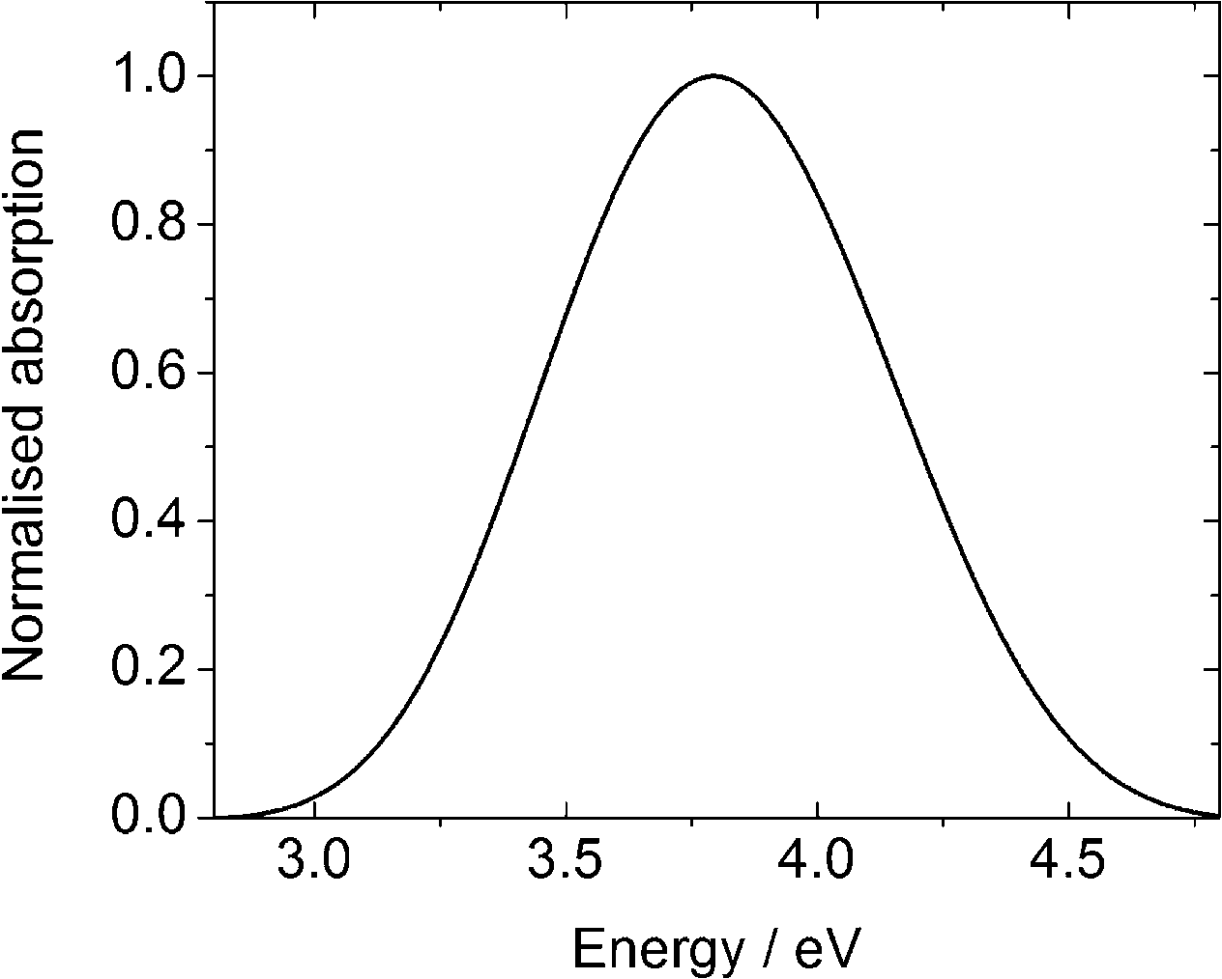
The absorption spectrum calculated by Fourier transformation of the autocorrelation function obtained from wavepacket propagation in the 2 ^1^A′ state of ClNO. The peak is at an energy of 3.79 eV, equivalent to 327 nm.

The autocorrelation function for this system is very simple: the wavepacket moves away from its initial position very rapidly and there are no recurrences. This results in a broad, featureless absorption spectrum peaking at an energy of 3.79 eV (equivalent to 327 nm), as shown in Figure 7, which can be compared to the experimental spectrum of Goodeve and Katz[10] and the calculated spectrum of Yamashita and Kato.[15]

Further information on rotational and vibrational product state distributions of NO can be obtained by considering the quantum flux passing into the CAP, as discussed above. For the NO vibrational states it was first necessary to calculate the lone NO potential-energy curve at the same level of theory as the ClNO PESs. The ab initio points were then fitted to an extended Rydberg function of the form [Eq. (10)]:

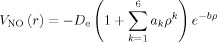
(10)
where *ρ*=*r*−*r*_e_. The value for *r*_e_ thus obtained matches the experimental value of 2.18 bohr, and the value of 6.49 eV for the dissociation energy *D*_e_ is nearly identical to the experimental value of 6.48 eV.[42] From this function the NO vibrational eigenstates could be found and then projected onto the asymptotic flux.

**Figure 8:**
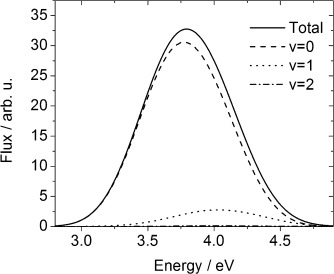
Energy distributions of the total quantum flux and that going into each product NO vibrational state obtained from a wavepacket propagation on the 2 ^1^A′ state of ClNO. The distributions are broad and featureless, with the peaks shifted slightly in energy for each vibrational state. The majority of NO is formed in *v*=0.

Figure 8 shows the flux passing into each NO vibrational state, and Table 1 lists the proportion of the total flux going into each state for the whole range of energies contained in the initial wavepacket. The total flux distribution is, as would be expected, broad and featureless like the absorption spectrum (Figure 7). The flux distributions into the individual vibrational states are similarly shaped, with the peaks shifted slightly in energy. The vast majority of NO is formed in *v*=0, with only 8 % in *v*=1. The populations of higher excited states are negligible. The photodissociation process is therefore essentially adiabatic with respect to the NO stretch.

**Table 1.** Fraction of the total flux going into each vibrational state of the NO product.

*v*(NO)	Percentage of total	
	with TDMs	without TDMs
0	91.2	78.9
1	8.3	17.4
2	0.38	3.1
3	0.11	0.53
4	0.04	0.09

Interestingly, if TDMs are not employed in the initial excitation and the relaxed wavepacket is simply placed “as-is” on the 2 ^1^A′ state, the calculated NO vibrational distribution is different. As shown in Table 1, although most NO is still formed in *v*=0, the proportion in *v*=1 is more than doubled. This is because the TDM operator has the effect of shifting more wavepacket density closer to the minimum in the *r*_NO_ coordinate, and hence gives less impetus in the direction of longer *r*_NO_.

To find the NO rotational state populations, the quantum flux was projected onto the spherical harmonics. This gives the flux distribution for each value of *j*, the NO rotational quantum number. Integrating these across all energies gives the overall NO rotational distribution, which is narrow and Gaussian-shaped. The peak is at *j*=51 and the full width at half-maximum (FWHM) is Δ*j*≈15. The product state distributions for a single energy, such as that for photodissociation with a nanosecond laser pulse, can be obtained from this one calculation by just looking at the flux results at that specific energy. Note that, unlike those for NO vibration, the rotational distributions are unchanged by inclusion of the TDM operator in the calculation.

### 2.3. Imaging Results

**Figure 9:**
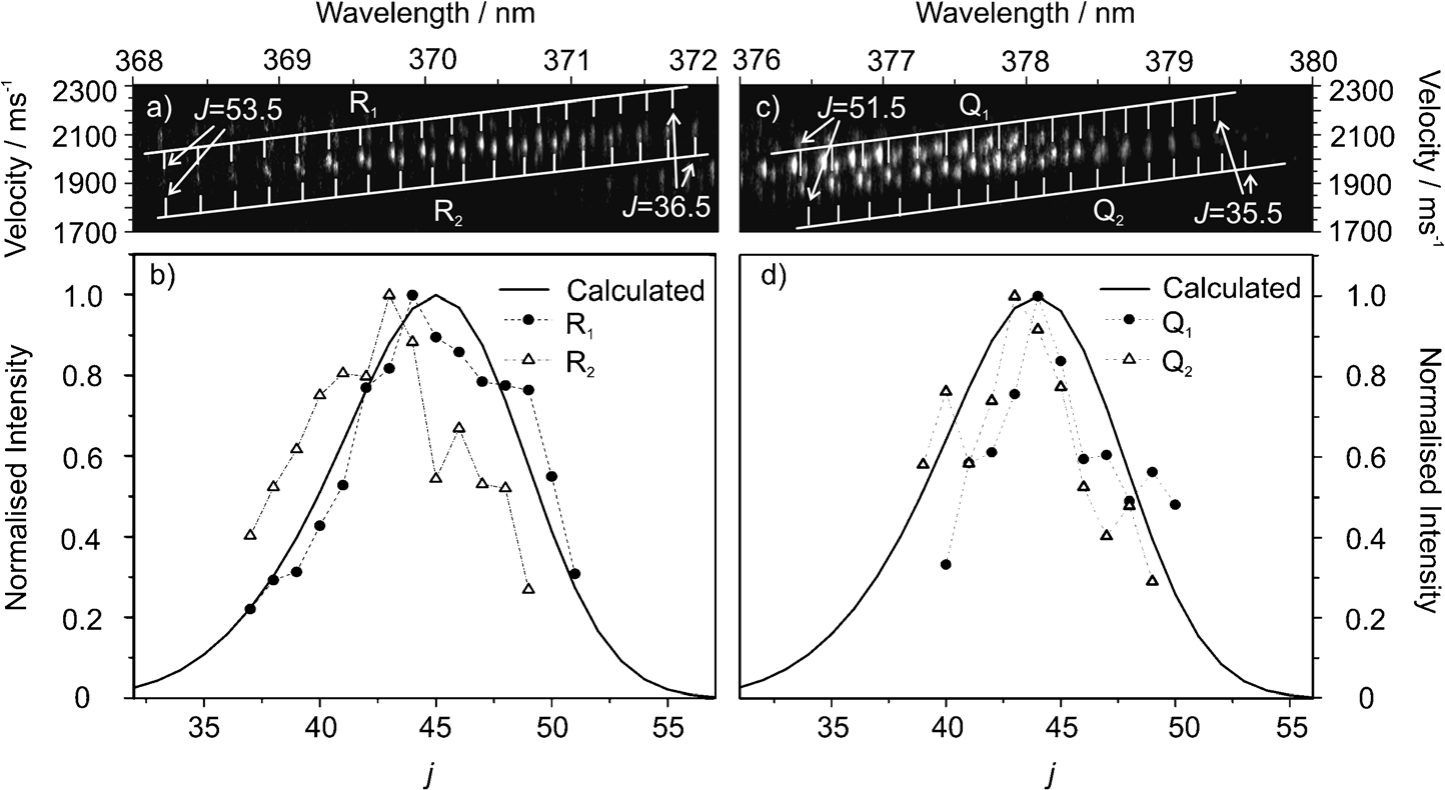
3D REMPI spectra and comparison of calculated and experimental NO rotational distributions. a) Experimental map of wavelength, photofragment velocity and intensity across the range 368.9–371.5 nm, which spans the R_1_ and R_2_ branches of the two-photon D←X transition in NO. Rotational combs are overlaid and show the assigned value of *J* for each peak, the intensities of which are extracted and presented in b), in which normalised NO rotational distributions for both the R_1_ (circles) and R_2_ (triangles) branches are compared with the calculated distribution (solid line). c) Spectrum for the wavelength region including the Q_1_ and Q_2_ branches of the C←X transition in the range 376.6–378.7 nm. d) NO rotational distributions extracted from each of the peaks in the assigned Q_1_ (circles) and Q_2_ (triangles) branches, compared with the calculated distribution (solid line).

A full report on our “3D slice imaging” results will be published elsewhere. Here a subset of the data is presented in the form of velocity-resolved REMPI spectra recorded between 369 and 379 nm, where the laser wavelength spans a number of (two-photon) rotational transitions in the D^2^Σ←X^2^Π (0,0) and C^2^Π←X^2^Π (0,0) bands (Figure 9 a and c). The spectra consist of several shorter scans which have been “stitched” together by normalising each scan to its neighbour by means of an overlap of 0.1–0.5 nm.

To assign the 3D REMPI spectrum, the wavelengths of each of the possible rotationally resolved REMPI transitions were calculated from the well-known spectroscopic constants of the D^2^Σ, C^2^Π and X^2^Π states of NO.^[^^43^^,^ ^44^^]^ Once the quantum state of each transition was known, its expected velocity could be calculated [for NO(*v*=0)] by using Equation (11):

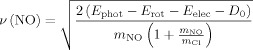
(11)
where *v*(NO) is the velocity of the NO photofragment formed in a particular quantum state, *E*_phot_ the photon energy, *E*_rot_ the rotational energy of the NO fragment, *E*_elec_ the spin–orbit electronic energy of the NO and Cl fragments, *D*_0_ the bond dissociation energy and *m*_Cl_ and *m*_NO_ are the masses of the Cl and NO fragments, respectively. The data represented in this form closely resemble a Fortrat diagram, for which a simple linear transformation is sufficient to convert the vertical axis of the spectrum from velocity to total angular momentum (*J*) space.

As is evident from Figure 9 a and 9 c, the agreement between the measured and predicted speeds of the NO photofragments from ClNO is very good. Moreover, the NO fragments are produced rotationally “hot” and in a narrow distribution. From the intensities of the peaks in the speed map the rotational distributions can be extracted by applying appropriate Hönl–London factors for each of the branches to correct for the relative line strengths. These are calculated by using PGopher[45] with a pure T[2,1] transition moment tensor for the D-X lines, and appropriate contributions from the T[0,0], T[2,0] and T[2,2] tensors for the C-X lines.[47]

As this is a one-colour experiment the different rotational branches are probed at slightly different excitation, and hence photolysis, wavelengths. The branches for the C and D states of NO are therefore considered separately. The C state is predissociative and hence gives rise to larger line widths than the D state.

The experiment yields rotational distributions in terms of *J*, the total angular momentum quantum number. For direct comparison with the computational results, these were converted to give distributions in terms of *j*. To achieve this, Hund’s coupling case (b) was assumed, as the spin–orbit coupling constant for NO is small (123 cm^−1^)[46] and the degree of rotational excitation is high.

The R_1_ and R_2_ branches of the D←X transition in NO were probed between 368.9 and 371.5 nm (3.34 to 3.36 eV). Rotational distributions for both spin–orbit states of NO are shown in Figure 9 b, in which those from the R_1_ branch (^2^Π_1/2_) are marked by circles, and those from the R_2_ branch (^2^Π_3/2_) by triangles. Both distributions have similar FWHM values of about Δ*j*=9, with the R_1_ distribution centred at *j*=46 and the R_2_ distribution centred a little lower at *j*=44. These results are overlaid on the calculated distribution for this small energy range (solid line in Figure 9 b). The calculated distribution peaks at *j*=45 and has an FWHM of Δ*j*=9.5.

The distributions resulting from the *Q*_1_ (^2^Π_1/2_, circles) and *Q*_2_ (^2^Π_3/2_, triangles) branches of the C←X transition between 376.6 and 378.7 nm (3.27 and 3.29 eV) are plotted in Figure 9 d. Because of the predissociative nature of the C state, the distributions are noisier than those for the D state but still exhibit similar features. The widths of the distributions are similar to those for the D state (Δ*j*≈8–9), and the *Q*_2_ distribution is shifted a little lower than that for the Q_1_ branch, centred at *j*≈44 as opposed to *j*≈45. The calculated distribution for the C-state energy range (solid line in Figure 9 d) has a peak at *j*=44 and an FWHM of Δ_*j*_=9.5.

## 3. Discussion

Experimental and computational rotational distributions of NO were presented in Figure 9. Despite some noise in the experimental distributions, the agreement between them is very good. The theory recreates the overall shape and widths of the distributions well, with the maxima of the rotational distributions agreeing to within 1–2 quanta. The energy difference between the C and D state transitions is small, but nonetheless appears to cause a shift in the NO rotational distribution of about one quantum towards higher *j* for the shorter-wavelength D state. The signal-to-noise ratio in the experimental results is not sufficient to confirm this effect, but the corresponding shift in the calculated distributions suggests that it is real. In addition, the experimental results suggest that the rotational distributions arising from the excited spin–orbit state of NO, ^2^Π_3/2_, are shifted by 1–2 quanta of *j* higher than those from its ground ^2^Π_1/2_ state, an observation that may be qualitatively explained on energetic grounds.

Previous studies on the photodissociation of the 2 ^1^A′ state of ClNO have been carried out at the slightly shorter wavelength of 355 nm. Our computational results at the equivalent energy predict an NO rotational distribution that peaks at *j*=47 with a shape and FWHM very similar to the distributions presented above. This is in good agreement with the distribution measured by Torres, Pipes and Baugh.[16] The calculation also reproduces the shape of the distribution obtained by Reisler and co-workers, although the calculated peak is a few quanta higher than the measured value.[9] There is very little difference in the distributions in the two NO spin–orbit states in the observations reported by Torres et al., whereas the results reported by Reisler and co-workers appear to show a slight shift of the NO ^2^Π_3/2_ distribution to lower *j*, which is similar to our observations.

## 4. Conclusions

New, purely ab initio PESs for the 1 and 2 ^1^A′ states of ClNO have been calculated. No additional parameters were added to artificially improve the fit to experiment. Wavepacket dynamics calculations modelling dissociation on the 2 ^1^A′ surface, incorporating the full transition dipole moment function, were run by using the MCTDH method. By analysing the quantum flux passing into a CAP placed in the asymptotic region of the PES, the expected vibrational and rotational product distributions in the NO channel were calculated. NO is produced almost exclusively in its ground vibrational state. However, the degree of vibrational excitation does depend weakly on the TDM function and is overestimated if this is neglected. The rotational distributions NO were found to be narrow and peaked at high *j*.

Three-dimensional REMPI experiments at photodissociation energies around 370 nm probed the NO product state distributions via the C←X and D←X bands of NO. As a consequence experimental NO rotational distributions were obtained in two slightly different wavelength regions. The computational NO distributions agree well with experimental distributions from both us and others, confirming that the computational model is a good one.
